# Effect of fermentation stillage of food waste on bioelectricity production and microbial community structure in microbial fuel cells

**DOI:** 10.1098/rsos.180457

**Published:** 2018-09-05

**Authors:** Hongzhi Ma, Cheng Peng, Yan Jia, Qunhui Wang, Maobing Tu, Ming Gao

**Affiliations:** 1 Department of Environmental Engineering, University of Science and Technology Beijing, Beijing 100083, People's Republic of China; 2 Beijing Key Laboratory of Resource-oriented Treatment of Industrial Pollutants, Beijing 100083, People's Republic of China; 3 Department of Biomedical, Chemical and Environmental Engineering, University of Cincinnati, 2901 Woodside Drive, Cincinnati, OH 45221, USA

**Keywords:** single-chamber microbial fuel cells, stillage, food waste, microbial community structure

## Abstract

A single-chamber microbial fuel cell (MFC) was used in this study to treat recycled stillage obtained from food waste ethanol fermentation. Corresponding substrates inside the system were evaluated by fluorescence spectra, and microbial communities were also investigated. Results demonstrated that output voltage and current, respectively, reached 0.29 V and 1.4 mA with an external resistance of 200 Ω. Corresponding total organic carbon and chemical oxygen demand removal efficiency reached more than 50% and 70%, respectively. Results of fluorescence spectra demonstrated that tryptophan-like aromatic, soluble microbial by-product-like and humic acid-like substances accumulated and were not easily degraded. Microbial community analysis by high-throughput sequence indicated that *Advenella* and *Moheibacter* occupied the highest proportion among all genera at the anode instead of *Geobacter*. These results may be due to complicated accumulated stillage, and potential tetracyclines possibly influenced microbial communities. Details on how stillage affects MFC operation should be further studied, and a solution on relieving effects should be established.

## 1. Introduction

Sustainability depends on access to energy services, which in the future will increasingly rely on renewable and alternative energy sources. As a renewable source of clean energy, fuel ethanol bears strategic significance in relieving shortage of petroleum resources and reducing environmental pollution [1]. Ethanol fermentation yields high volumes of stillage, which entails unconvertible organic fractions, low pH and high percentage of dissolved organic and inorganic matters [2,3]. Characterized with high volume and chemical oxygen demand (COD), difficulty arises from managing stillage. As reported, 10 tons of wastewater, including stillage and other wastewater caused by washing or cleaning, are produced during production of 1 ton of ethanol; this condition became a serious challenge for industrial development of fuel ethanol [4,5]. Stillage recycling is a simple and economical method of solving stillage pollution. However, stillage reflux cannot be limitless. After several times of refluxing, stillage composition becomes complicated and must be dealt with [6].

Composting represents a widely used and effective method for converting organic wastes into relatively stable humus-like product that can be used as soil amendment or organic fertilizer. It simultaneously combines material recycling and biomass disposal and allows the conversion of biodegradable organic wastes (such as agricultural leaves, sewage sludge and food waste) into stabilized end products [7,8]. But, some major drawbacks associated with composting are nitrogen (N) loss and greenhouse gas (GHG) emissions. Especially, manure composting has been universally regarded as a source of anthropogenic GHG such as carbon dioxide (CO_2_), methane (CH_4_) and nitrous oxide (N_2_O) [7].

One of the revived bio-electrochemical concept and promising technology that is proposed to address these aspects is microbial fuel cell (MFC), which principally produces electricity from the anaerobic oxidation of biodegradable organic substrates [9].

MFC technology offers potential as alternative to the present biological wastewater treatment systems because of its ability to transform energy-consuming processes into energy-saving ones [10–13]. MFC is a device that uses bacteria to digest organic matters and generates current. Growth of organic matter-decomposing microorganisms results in the release of protons and electrons at the anode electrode. Electrons and protons are separately transferred from anode to cathode through external circuits and proton transfer systems. Therefore, circuits close, and electricity is produced. The electricity generation with various simple molecules as electron donors has been tested over the years. The use of pure substrates has been the choice of researchers focusing on different fundamental aspects of MFCs operating mainly with pure cultures. Meanwhile, attributed to the ability of MFCs for wastewater treatment with simultaneous energy recovery, several waste streams have been tried as substrates so far [9]. Hui Li *et al.* used MFCs to recover electricity from canteen-based food waste. A maximum power density of 5.6 W m^−3^ and average output voltage of 0.51 V were obtained [14]. Stillage after refluxing for several times consists of high amounts of volatile fatty acids and lactic acid, which can be easily degraded by microorganisms in MFCs. Salts in stillage can improve electrical conductivity, which is advantageous for proton transfer. Stillage can be an appropriate substrate for MFCs. Sakdaronnarong *et al.* investigated feasibility of using lignin waste from pulp and paper industries as mediator for treating ethanol stillage wastewater in two-chamber MFCs. In their study, 93 W m^−2^ with COD removal efficiency of 81% was obtained. This previous study simultaneously showed potential for treatment of lignin-rich wastewater and ethanol stillage wastewater with the possibility of electric power generation [15]. However, little information is available on the use of single-chamber MFCs in food waste ethanol stillage.

According to configuration, MFCs can be divided into single-chamber and two-chamber MFCs. A two-chamber MFC consists of anode and cathode compartments separated by a proton exchange membrane [16]. Oxidation-reduction reactions occur in different compartments. The protons generated in the anode compartment transfer through proton exchange membrane to the cathode compartment, and electrons transfer through the external circuit. Existence of proton exchange membrane can improve coulombic efficiency (CE) of MFCs and provide a strict anaerobic environment for microorganisms [15,17]. However, the proton exchange membrane can increase internal resistance, thereby reducing power generation. Expensive cost is also unsuitable for large-scale application of MFCs. Single-chamber MFCs comprise one anode compartment, in which electrochemical reaction occurs, whereas the cathode is exposed to air. Single-chamber MFCs feature advantages, including low resistance, low cost and simplified configuration. Oxygen is used as an oxidizing agent for clean production of water at the cathode. Single-chamber MFCs perform better than two-chamber MFCs but cannot provide strict anaerobic environment, in which oxygen diffusion may affect growth of exoelectrogenic bacteria. Microorganisms may be flushed away from the anode and form biofilms on the cathode in single-chamber MFCs. Microbial community structures in MFCs were extensively researched. Jianna Jia *et al.* used food wastes as substrates in MFCs. A maximum power density of approximately 18 W m^−3^ (approx. 556 mW m^−2^) was obtained at COD of 3200 ± 400 mg l^−1^, and maximum CE reached approximately 27.0% at COD of 4900 ± 350 mg l^−1^. In their study, the majority of dominant populations belonged to *Geobacter* (37.72%) and *Bacteroides* (34.66%). *Geobacter* occupied the highest proportion among all genera and played a key role in power generation [18]. However, no research currently studies microbial community structure in MFCs with stillage as substrate.

In this study, a single-chamber MFC was used to treat food waste ethanol fermentation stillage after several reflux times. Electricity recovery from stillage and its influence on the system were also measured; stillage influence includes substrate removal and microbial community structure on both electrodes.

## 2. Material and methods

### 2.1. Stillage as culture medium

Food waste after pretreatment was used for ethanol fermentation as described in a previous article [19]. Fermentation broth discharged from the fermenter was collected and distilled to remove ethanol and volatile by-products. The remaining stillage with non-volatile by-products was recycled for saccharification and broth preparation [6]. After five times of reflux, stillage was collected and stored in a refrigerator. The medium contained the following (grams in 1 l deionized water): KCl, 0.26; NaH_2_PO_4_ · 2H_2_O, 5.54; Na_2_HPO_4_ · 12H_2_O, 23.08; and NH_4_Cl, 0.62, as reported in [20]. Diluted stillage with COD of 700 mg l^−1^ was fed as substrate and the phosphate buffer solution (PBS, 50 mM, pH 7.0) was used.

### 2.2. Microbial fuel cell configuration and operation

A single-chamber air cathode MFC with a working volume of 120 ml was constructed (the type is shown in electronic supplementary material, S1). Spacing between the anode and cathode placed on opposite sides measured 4 cm. The anode was made of graphite felt (40 × 40 mm; 5 mm thickness) and subjected to certain pretreatment. Graphite felt was soaked in acetone and ethanol successively and then washed with deionized water. The air cathode with an area measuring 12.56 cm^2^ consisted of a conductive gas diffusion layer and a catalyst layer, with titanium wire as the current collector. The conductive gas diffusion layer supplied air to the catalyst layer and prevented leakage of liquid electrolytes. The hydrophobic conductive gas diffusion layer was prepared by blending carbon black and polytetrafluoroethylene (PTFE) with a mass ratio of 2 : 3. Mixture was rolled into a membrane and then sintered for 30 min at 320°C to melt PTFE to form fibrous three-dimensional structure for gas transport. Then, 40% Pt/C catalyst (0.5 mg Pt cm^−2^) was applied to one side (water-facing side) of carbon membrane by using Nafion as the binder.

MFC was inoculated with the solution from a laboratory-scale microbial electrolysis cell reactor; stillage after dilution was directly used as substrate. External resistance connected across the anode and cathode was 200 Ω. MFC was repeatedly filled with inoculant and substrate until a constant output voltage was obtained. The MFC fed with stillage was operated in batch mode at room temperature and refilled when output voltage decreased to lower than 100 mV, thereby forming an operation cycle.

### 2.3. Calculations and analyses

Output voltage (V) and current (mA) were measured by a battery testing system (CT-3008-5V30 mA-S4). To obtain polarization and power density curves as a function of current, an electrochemical workstation (CHI660E B14665a) was used to test linear sweep voltammetry (LSV) of the whole cell. Power (W or mW) was calculated according to the formula *P* = *U* × *I*, where *P* is the power density (W), *U* is the voltage (V) and I is the current (A). Power density (mW m^−2^ or W m^−3^) was calculated by dividing output power with cathode area (m^2^) or chamber volume (m^3^). Influent and effluent samples were collected during operation. Cyclic voltammetry (CV) tests were conducted on the bioanode within the potential range of −0.6 to 0.3 V at a scanning rate of 0.01 V s^−1^ versus Ag/AgCl. After centrifugation for 12 min at 14 000 r.p.m., the supernatant was subjected to testing of total organic carbon (TOC) and COD. TOC was measured using a TOC analyser (Vario TOC). COD was measured according to standard methods. For testing lactic acid, samples went through a 0.45 µm membrane filter after centrifugation. Lactic acid was measured using biosensor SBA-40C.

### 2.4. Fluorescence spectroscopic analysis

Influent and effluent samples were centrifuged at 14 000 r.p.m. for 12 min. Excitation-emission matrix (EEM) spectra were monitored using a F-2700 FL spectrophotometer by scanning excitation spectra from 220 to 400 nm with increments of 5 nm, detecting emission wavelength between 280 and 550 nm with 5 nm steps. Slits were maintained at 5 nm for both excitation and emission, and scanning speed measured 1500 nm min^−1^.

### 2.5. DNA extraction, polymerase chain reaction amplification and Illumina MiSeq sequencing

Samples were collected from the graphite felt anode and carbon membrane cathode of MFC, which was under stable operation conditions for more than one month. Microbial DNA was extracted from electrode samples by using the E.Z.N.A.® DNA Kit (Omega Bio-tek, Norcross, GA, USA) according to manufacturer's protocols.

The V4–V5 regions (length of approx. 392 bp) of bacterial 16S rRNA gene were amplified by PCR using primers 515F 5′-barcode-GTGCCAGCMGCCGCGG)-3′ and 907R 5′-CCGTCAATTCMTTTRAGTTT-3′, where the barcode is an eight-base sequence unique to each sample. Purified amplicons were pooled in equimolar and paired-end sequenced (2 × 250) on an Illumina MiSeq platform according to standard protocols.

Operational units were clustered with 97% similarity cut-off by using UPARSE (v. 7.1 http://drive5.com/uparse/), and chimeric sequences were identified and removed using UCHIME. Taxonomy of each 16S rRNA gene sequence was analysed by Ribosomal Database Project Classifier (http://rdp.cme.msu.edu/) against the silva (SSU115)16S rRNA database at a confidence threshold of 70%. Corresponding data can be seen in electronic supplementary material, S2.

## 3. Results and discussions

### 3.1. Bioelectricity generation of the stillage-microbial fuel cell

**Figure 1.**
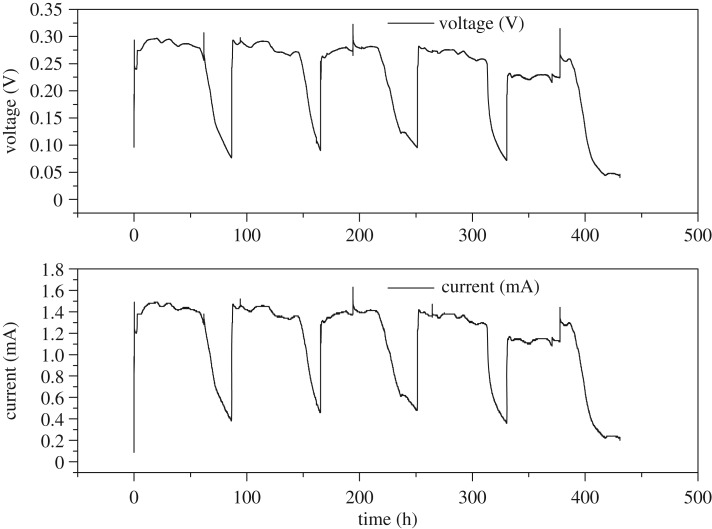
Variations in voltage and current for MFC-treated stillage (with external resistance of 200 Ω).

After acclimation period of 5 days, stable electricity generation was achieved in the MFC. As shown in figure 1, voltage and current increased rapidly after stillage was fed into MFC. Output voltage and current reached approximately 0.29 V and 1.4 mA, respectively, with an external resistance of 200 Ω, and then remained stable for 50 h. Then, output voltage and current decreased gradually because easily biodegradable components in the substrate were used up. Then, components that were difficult to degrade were used to produce electricity.

**Figure 2.**
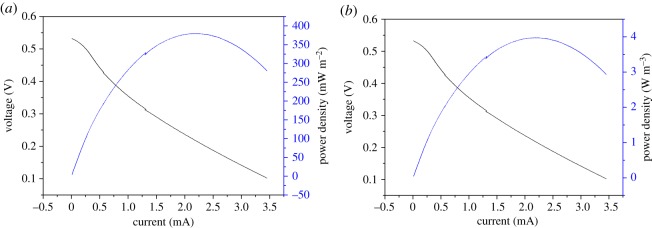
Variation in polarization curves and power density for MFC (*a*) power density based on area of the cathode (*b*) power density based on the reactor volume.

The polarization curve was obtained using the electrochemical workstation scanning LSV curve. The cathode was used as the working electrode, and the anode was used as the counter and reference electrode. Maximum power density of MFCs can be obtained from the polarization curve. Open-circuit voltage of MFC was measured as 0.55 V using an electrochemical workstation, and this value was lower than those of MFCs with pure substances as substrates. This finding is due to complex composition of ethanol fermentation stillage from food waste used as substrate. As shown in figure 2, maximum power density obtained measured 379.4 mW m^−2^ based on cathode area and 3.97 W m^−3^ based on the reactor volume. Jianna Jia *et al.* used a single-chamber air cathode MFC with 28 ml volume to treat food waste and obtain a maximum power density of approximately 18 W m^−3^ (approx. 556 mW m^−2^) [18]; this result was probably due to differences in substrate. Composition of stillage can be more complicated than food waste, which is not easily degraded by MFC. Magnification of reactors can result in decrease in power density. Several studies focused on MFCs with stillage as substrates. Sakdaronnarong *et al.* used a two-chamber MFC to treat stillage. A power density of 93 W m^−2^ was obtained with COD removal efficiency of 81% [15,21]. A maximum power density of 1180 mW m^−2^ was obtained with post-fermentation biorefinery stream as substrate in an air-cathode MFC [22]. Compared with values obtained in studies with stillage as substrates, power density obtained in our study was relatively low. After several reflux times, composition of stillage became complex. Several macromolecular substances in post-treatment stillage were difficult for the MFC to use and caused inhibition of microorganisms in the system. However, Jooyoun *et al.* used MFC to treat real fermented wastewater and generated a maximum power density of 2981 mW m^−3^ [23], which is similar to our power density. Therefore, we can consider that the power obtained in our experiment was within the normal range, indicating that we successfully recovered electricity from stillage by using MFCs.

### 3.2. Cyclic voltammogram

**Figure 3.**
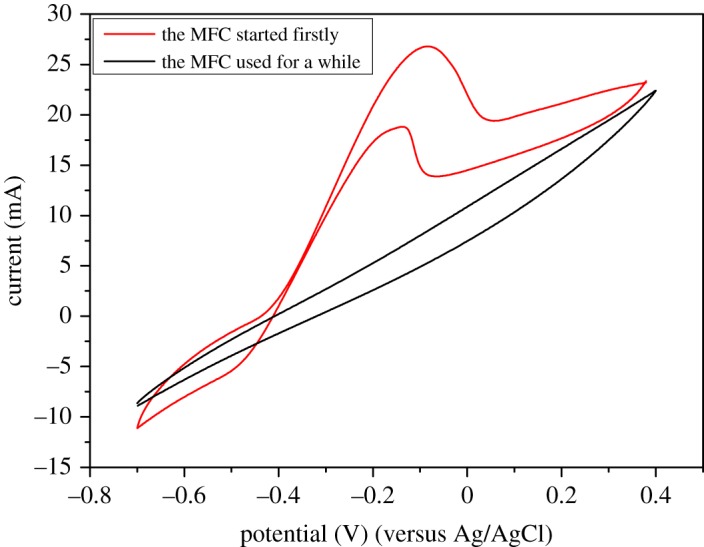
Cyclic voltammograms.

CV was performed on a stable bioanode. Cyclic voltammograms were obtained immediately after the replacement of a fresh substrate. As shown in figure 3, the red line indicates that MFC was successfully started first, and the black line shows that performance decreased after a certain period. Significant oxidation and reduction peaks were observed when operation was started. A peak was observed at −0.082 V, and an additional peak was detected at −0.065 V, showing that the current increased suddenly at these two potentials. This result indicated electrochemical activity of the anode. The figure shows good electrochemical activity at the beginning. After operation for a certain period, electrochemical activity evidently decreased. In Jooyoun *et al.*'s study, affinity of electrochemically active bacteria strongly depended on the availability of substrates [23]. In our study, availability status of stillage was not ideal. Reactants were available in the initial period of substrate supplement. Complex organics were converted into simple organics upon consumption of original simple organics. Other unavailable complex organics recognized as side-products were probably generated in this process. Complex non-biodegradable components accumulated and possibly caused inhibition of microorganisms in the system. Therefore, electrochemical activity decreased after a certain period.

### 3.3. Removal rates

**Figure 4.**
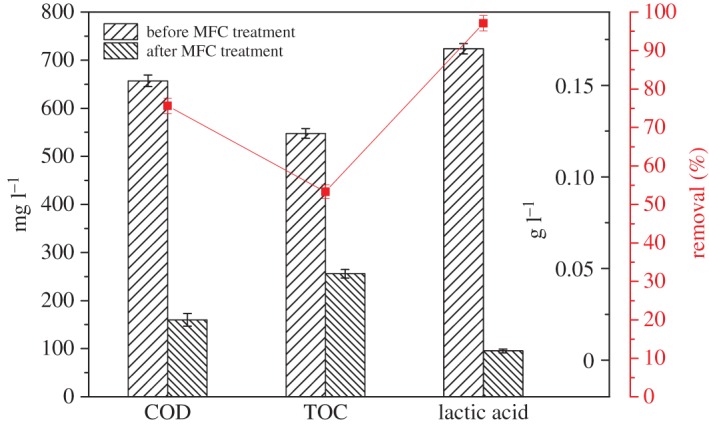
Removal rates of organics.

Using wastes as substrates, MFCs were used to recover electric energy. High removal rate and high energy output were required. Thus, our research studied removal rates of COD, TOC and lactic acid. Lactic acid was selected because Ma Hongzhi *et al.*'s study indicated that accumulation of lactic acid and salts caused inhibition of ethanol production during stillage reflux [24]. In this case, lactic acid in stillage was practically a side-product. Figure 4 shows removal rates of COD, TOC and lactic acid. Lactic acid was almost completely removed after treatment of MFC, and removal rate reached 97.1% as these compounds were easily degraded by MFCs. However, removal rates of COD and TOC in several batches totalled 75.6% and 53.3% on the average, respectively. In other studies, removal rates of COD reached 70%–80% or exceeded 80% [15,18,23]. Results of our experiment are correspondingly low. In Vogl *et al.*'s study, MFCs with idealized blackwater displayed stable power densities in batch operation, with TOC removal of 64% ± 9% [25], which is higher than the TOC removal rate in our study. Results differed because of varying substrates used. Our study showed relatively good and low removal rates of COD and TOC, respectively. Before MFC treatment, there are many reducing substances in the stillage, including some reducing inorganic substances, which makes the content of COD exceed TOC. After the electrochemical treatment of MFC, some of the macromolecular substances in the stillage are broken down into small molecular substances, some of which cannot be oxidized and reduced. These findings indicate that stillage composition, which can be oxidized by potassium dichromate, can be also degraded by MFCs. However, several complex organics present in stillage cannot be oxidized by oxidizing agents. These organics are difficult or impossible to biodegrade by microorganisms in MFCs. Stillage composition is complex. Non-biodegradable components accumulated during stillage reflux and cannot be used by MFCs, possibly causing inhibition of growth of microorganisms in the system.

### 3.4. Fluorescence excitation-emission matrixs analysis

**Figure 5.**
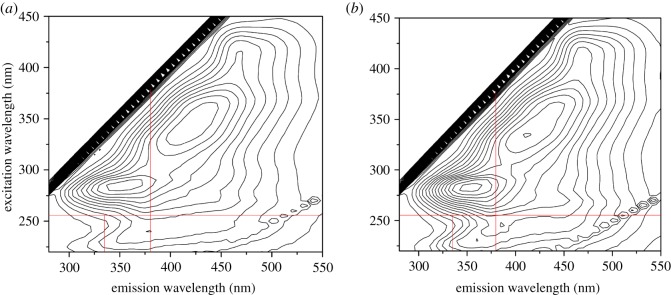
Fluorescence EEMs for influent and effluent of the MFC (*a*) influent, (*b*) effluent.

To study changes in other compositions, fluorescence EEMs were conducted on influent and effluent of MFC. Figure 5 shows corresponding fluorescence intensity contour plots.

As shown in figure 5, both EEMs of influent and effluent presented four peaks and similar positions, respectively. According to a previous study [26], Peak 1 (Ex/Em 240/380) represented tryptophan-like aromatic substances, Peak 2 (Ex/Em 285/355) indicated soluble microbial by-product-like substances, and Peaks 3 (Ex/Em 335/410) and 4 (Ex/Em 360/440) denoted humic acid-like substances. Table 1 shows positions and corresponding intensities of fluorescence peaks. Intensity of these four fluorescence peaks increased by 44.6%, 32.5%, 11.5% and 8.3%, respectively. Tryptophan-like aromatic, soluble microbial by-product-like and humic acid-like substances in substrate were not easily degraded by MFCs or may be intermediate metabolites of several original components.

**Table 1.** Position and corresponding intensity of fluorescence peaks.

samples	Peak 1		Peak 2		Peak 3		Peak 4	
	*λ*_ex_/*λ*_em_ (nm)	*I*_1_	*λ*_ex_/*λ*_em_ (nm)	*I*_2_	*λ*_ex_/*λ*_em_ (nm)	*I*_3_	*λ*_ex_/*λ*_em_ (nm)	*I*_4_
influent	240/380	75.59	285/355	174.8	335/410	175.9	360/440	171.7
effluent	240/370	109.3	285/355	231.6	335/410	196.1	360/440	186.0

Corresponding fluorescence peaks of influent and effluent belong to the same organics, but specific positions in fluorescence spectra were not specifically identical. As shown in table 1, the fluorescence peak for tryptophan-like aromatic proteins exhibited a 10 nm blue-shift along the emission axis. Types of structural changes which can result in blue-shift emission include (i) reduction in extent of the π-electron system, for instance, decrease in the number of aromatic rings or conjugated bonds in chain structures or conversion of linear ring systems to nonlinear ones; and (ii) elimination of specific functional groups, such as carbonyl, hydroxyl and amine [27]. Therefore, dissociation of complex aromatic rings and large-particle organics into small fragments occurred during treatment of MFCs. However, generated products possibly posed difficulty in degradation by MFC and therefore accumulated in the system.

### 3.5. Microbial community structure of anode and cathode biofilms

As mentioned above, biodegradable components accumulated during MFC treatment with stillage as substrates, including the original complex components and intermediate metabolites generated. This accumulation possibly affected growth of microorganisms in the system. Sequencing was conducted on the anode and cathode biofilms to study microbial community structure with stillage as substrates. In single-chamber MFCs, the anode and cathode are in one compartment without a proton exchange membrane. Thus, microorganisms may be flushed away from the anode and form a biofilm on the cathode. Power density obtained in our study was comparatively poorer than those in other studies. It was found in previous studies that with the gradual accumulation of attached biomass over long periods of operation, some operation problems eventually emerge in biofilters. Uneven biomass accumulation and consequent biological clogging are usually considered to be among the major problems in the media of gas phase biofilters [28,29]. Alonso *et al*. [30,31] consider that uneven biomass distribution leads to some operational problems including clogging, short-circuiting and increased pressure drop, and deteriorated removal efficiency, especially at high organic loading rates and for a long duration of operation of biofilters. Therefore, the complex composition of the stillage and the uneven distribution of microorganisms at the cathode and anode led to this result. High-throughput sequencing was conducted on the anode and cathode biofilms by Illumina MiSeq sequencing platform to analyse community structure and variation characteristics of microorganisms. The majority of dominant populations belonged to exoelectrogenic and fermentative bacteria.

**Figure 6.**
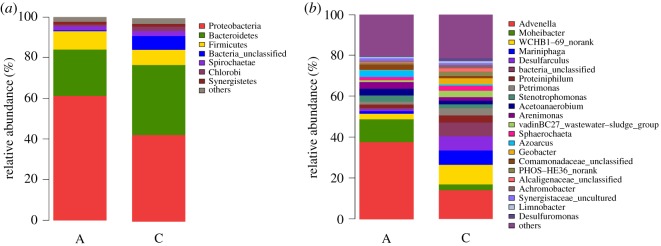
Microbial community structure of the anode and cathode biofilms (*a*) at phylum level (A, anode; C, cathode), (*b*) at genus level (A, anode; C, cathode).

As shown in figure 6*a*, the majority of dominant populations belonged to *Proteobacteria* (61.2% at the anode, 42.9% at the cathode), *Bacteroidetes* (22.9% at the anode, 34.5% at the cathode) and *Firmicutes* (9% at the anode, 7.5% at the cathode), accounting for more than 80% of total bacteria. *Proteobacteria* occupied the highest proportion among all phyla. Species of microbial community structure on the anode and cathode biofilms were the same, but their relative abundances differed. Inoculation was performed in the anode chamber, but a biofilm grew on the cathode. Thus, the MFC lost its microorganisms. Cathode biofilm originated from the anode. Relative abundance of *Proteobacteria* and *Firmicutes* on the anode biofilm was higher than that on the cathode biofilm, whereas that of *Bacteroidetes* on anode biofilm was lower than that on the cathode biofilm.

As shown in figure 6*b*, species of microorganisms at the genus level were analysed after the phylum level. The majority of genera on the anode film included *Advenella* (37.8%) and *Moheibacter* (11%), whereas that on the cathode film comprised *Advenella* (14.2%), *WCHB1-69* (9.7%), *Desulfarculus* (7%) and *Mariniphaga* (6.97%), and each of the bacteria was not markedly different.

*Advenella* is a type of mesophilic bacterium that can be separated during different composting processes. This bacterium is a new strain of tetracycline-degrading bacteria and can be obtained from pharmaceutical factory wastewater after separation and screening. *Advenella* was demonstrated to degrade tetracycline under suitable conditions of pH 7.0 and 30°C. After 6 days of culture, degradation rate of tetracycline reached 57.8% at an initial concentration of 50 µg ml^−1^. In our research, *Advenella* occupied the highest proportion among all genera on both anodes and cathodes. Tetracycline possibly exists in food waste and cannot be degraded. As Lee *et al.* discovered, tetracycline antibiotic-resistance gene widely exists in food waste-recycling wastewater [32]. Tetracycline also remains in stillage after fermentation and several times of reflux. Microorganisms in the MFC were screened by tetracycline during operation. The proportion of *Advenella* was extremely high relative to those of other bacteria, and its proportion on the anode was markedly higher than that on the cathode. This result was probably due to the pretreatment of anode materials to be hydrophilic, which benefited growth of microorganisms, whereas cathode materials were pretreated to be hydrophobic. Thus, microbial community structure on the anode differed from that on the cathode. *Moheibacter* was another high-content bacterium on the anode. *Moheibacter* is a Gram-negative, non-sliding bacillus that presents bright-yellow, round, smooth and mucoid colonies. Studies showed sensitivity of *Moheibacter* to tetracycline. Thus, *Moheibacter* grew well in the presence of *Advenella*. *Moheibacter* can grow under conditions of 4°C to 33°C, pH 6.0–10.0 and 0% to 3.0% (w/v) NaCl, with optimum growth conditions at 28°C, pH 7.0–7.5 and 0% to 0.5% (w/v) NaCl. Conditions with stillage as substrate were close to those for optimum growth, and these parameters caused difficulty in flushing away *Moheibacter*. Hence, the proportion of *Moheibacter* on the anode was markedly higher than that on the cathode.

Proportions of well-known exoelectrogenic bacterium *Geobacter* reached 0.3% and 3% on the anode and cathode, respectively. *Geobacter* was present at extremely low proportions on the anode biofilm and much lower proportions on the cathode biofilms. In Jianna Jia *et al.*'s study, *Geobacter* occupied the highest proportion (37.72%) among all genera [18], exceeding the proportion detected in our study. In our experiment, *Advenella* and *Geobacter* were present at high and extremely low proportions, respectively, probably because growth of *Advenella* exerted an inhibitory effect on *Geobacter*. Composition of stillage was complicated, and accumulation of harmful substances possibly induced inhibition. Stillage presents low oxidation reduction potential and therefore cannot produce an appropriate environment for *Geobacter* growth. These circumstances can explain poor reactor operations.

Microbial community structures were demonstrated to significantly induce manifestation of MFCs through high-throughput sequencing. However, further studies should explore mechanisms of how stillage compositions exert inhibitory effects on microbial community structures and how to eliminate such influences.

## 4. Conclusion

A single-chamber MFC was successfully used to simultaneously treat food waste ethanol fermentation stillage and recover electricity. Removal rates of COD and TOC exceeded 50% and 70%, respectively, and lactic acid removal reached 97.1%. Accumulation of components in stillage may exert inhibitory effects on growth of microorganisms. Tryptophan-like aromatic, soluble microbial by-product-like and humic acid-like substances in substrate accumulated and were difficultly degraded by MFCs. High-throughput sequencing indicated that microbial community structures were also influenced by complex substrates and that exoelectrogenic bacteria gradually became non-dominant strains.

## Data accessibility

This article does not contain any additional data.
